# Distinguishing drought resistance strategies and identifying indicator traits of *Platycladus orientalis* and *Broussonetia papyrifera*

**DOI:** 10.3389/fpls.2025.1644756

**Published:** 2025-08-20

**Authors:** Kai Yao, Chunxiu Tu, Aoli Zhang, Zhaoxia Zeng, Yang Yang

**Affiliations:** ^1^ School of Life Science, Guizhou Normal University, Guiyang, China; ^2^ Guizhou Key Laboratory of Forest Cultivation in Plateau Mountain, Guizhou Normal University, Guiyang, China; ^3^ Key Laboratory of Agro-Ecological Processes in Subtropical Region, Institute of Subtropical Agriculture, Chinese Academy of Sciences, Changsha, China; ^4^ Huanjiang Observation and Research Station for Karst Ecosystem, Chinese Academy of Sciences, Huanjiang, China

**Keywords:** drought resistance strategies, stomatal transpiration efficiency, karst plants, indicator trait, isohydric-anisohydric behavior

## Abstract

Tree species adopt diverse drought resistance strategies, which are crucial for the ability of karst vegetation to adapt to drought stress. However, our understanding of how to differentiate these strategies remains limited, particularly with respect to identifying indicator traits that can accurately distinguish the drought resistance strategies used by different species. In this study, we use principal component analysis based on functional traits to distinguish the drought resistance strategies of *Platycladus orientalis* and *Broussonetia papyrifera*; we identify key indicator traits reflecting differences in drought resistance strategies by analyzing the correlations of the same traits across different plant species. Most importantly, in this study, stomatal transpiration efficiency is proposed as a novel trait. Principal component analysis based on functional traits can distinguish plant drought resistance strategies. A correlation analysis of the indicators revealed that 2,2-diphenyl-picrylhydrazyl radical-scavenging activity, Δcrown width, stomatal transpiration efficiency, and water use efficiency can serve as critical markers to differentiate the drought resistance strategies of plants. Notably, the stomatal transpiration efficiency of *P. orientalis* and *B. papyrifera* exhibited entirely opposite trends under drought stress (*r* = -0.38); however, investigations of additional tree species are needed to further verify the reliability of stomatal transpiration efficiency as an indicator of different plant drought resistance strategies. These findings improve our ability to effectively differentiate karst plant drought resistance strategies and understand the mechanisms involved.

## 1 Introduction

The continuous increase in CO_2_ concentrations in the atmosphere due to human activities has led to a greenhouse effect that has unquestionably caused global warming (An et al., 2017). To date, the global temperature has risen by 1.5°C compared with that before the industrial revolution (IPCC, 2018). This change may lead to increases in the duration, intensity and frequency of droughts worldwide (Cramer et al., 2018) and will have a continuous impact on biodiversity and the stability of ecosystems globally. Thus, the adaptability and adaptation mechanisms of forest ecosystems during drought events have become major concerns for scientists (Allen et al., 2010; Lloret et al., 2012; Kramp et al., 2022). Species diversity is considered a key factor in determining the drought resistance of vegetation (Messier et al., 2022). In particular, the combination of species within a community that adopt different drought resistance strategies has an important impact on the resistance and dynamics of the community under drought stress (Forrester and Bauhus, 2016; Grossiord, 2020). Although plants in global karst regions possess unique lithophytic and xerophytic characteristics, we still know very little about how to distinguish the drought resistance strategies of karst plants, especially which indicator traits can be used to accurately distinguish the drought resistance strategies of plants. This information would improve our understanding of the drought resistance mechanisms of karst vegetation and aid in the prediction of vegetation succession under future climate conditions.

**Figure 1:**
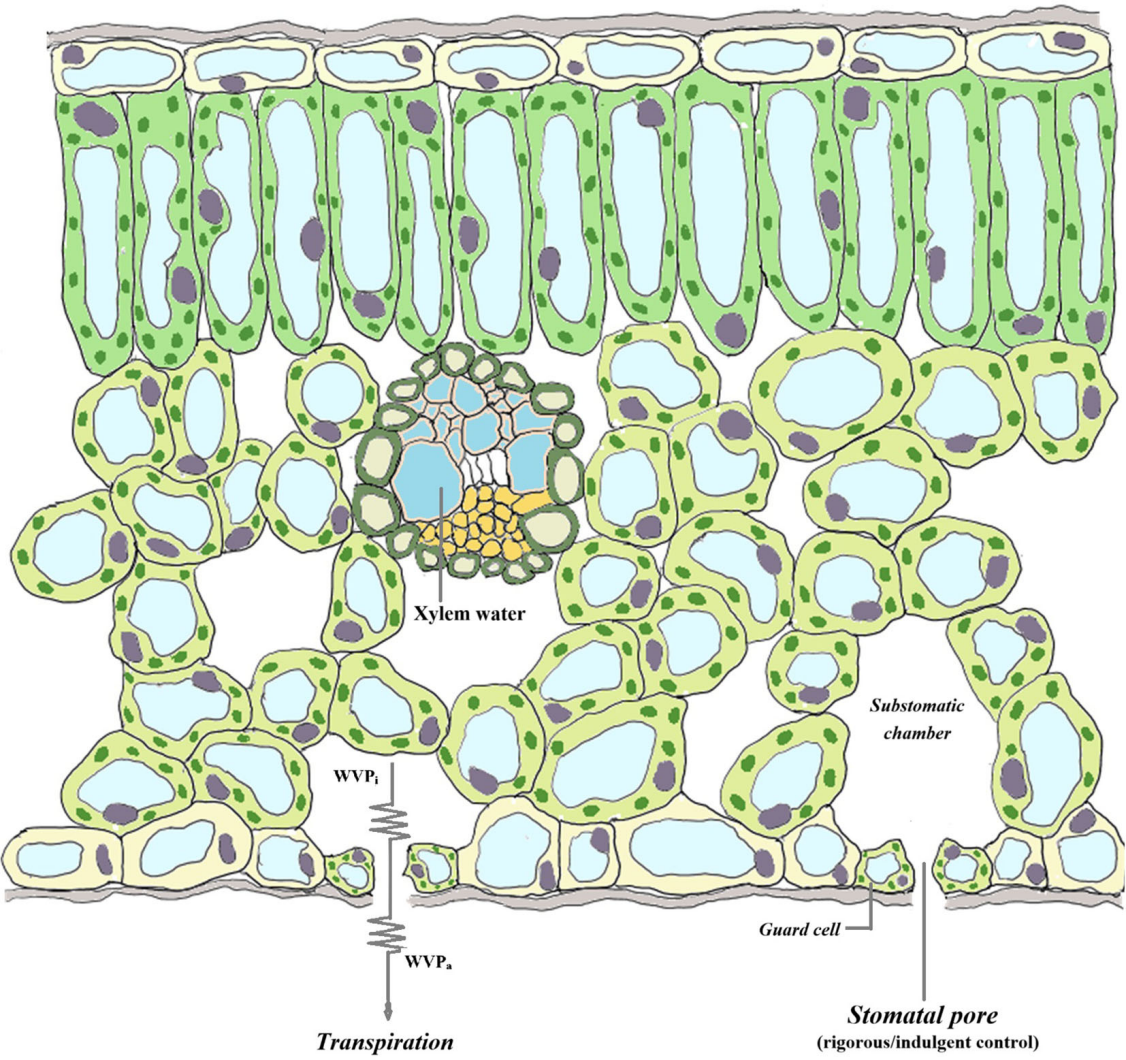
Water vapour diffusion and stomatal control in plants. The generation of leaf transpiration is due to the vapour pressure deficit (VPD) when the water vapour partial pressure in the air space beneath the stomata (WVP_i_) is greater than that in the air (WVP_a_). Plants usually replenish their internal water at night. However, under drought conditions, the degree of water recovery in anisohydric species before predawn is lower than that in isohydric species, coupled with their independent stomatal management strategies (i.e., a greater degree of stomatal opening). Therefore, compared with those of isohydric species, the water vapour partial pressure in the air space within the stomata of anisohydric species is lower, and the rate of water molecule diffusion out of the stomata is slower; that is, the number of water molecules passing through the stomatal cross-section per unit time is lower. On the basis of this assumption, we propose the concept of stomatal transpiration efficiency (STE).

Species-specific factors often lead to differences in the adaptive characteristics of different plants in response to drought stress. In addition to escape strategies, plants often adopt drought avoidance or drought tolerance strategies in arid environments. Drought avoidance strategies are most notably characterized by maintaining high tissue water content under drought conditions through increased water absorption by the root system and reduced transpiration from leaves. In contrast, drought tolerance strategies can minimize damage caused by dehydration through various physiological and morphological regulations, such as strong osmotic adjustment, peroxide scavenging, and cellular elasticity and protoplasmic resistance (Anderegg and Hillerislambers, 2016; Yildirim and Kaya, 2017; Kramp et al., 2022). Additionally, the isohydric–anisohydric spectrum model, which focuses on stomatal regulation behavior in drought environments, suggests significant differences in physiological regulation among plants adapting to drought conditions (Tardieu and Simonneau, 1998; Choat et al., 2012). Plants that adopt isohydric behavior under drought conditions rapidly close their stomata to maintain a certain water potential, and their photosynthetic rate also decreases, making them prone to carbon starvation; in contrast, plants that adopt anisohydric behavior can maintain relatively high stomatal opening under drought conditions to sustain a high photosynthetic rate, but they are often threatened by hydraulic imbalance (Jones, 1998; Tardieu and Simonneau, 1998; McDowell et al., 2008). On the basis of isohydric–anisohydric spectrum theory, we propose the concept of stomatal transpiration efficiency (STE) to better describe, by observations, the stomatal management strategies of plants under different environmental conditions. If plants adopt strict stomatal management measures and regulate stomata when the water content slightly changes, i.e., isohydric plants (Tyree and Sperry, 1988; Ulrich and Grossiord, 2023), their STE should remain relatively constant; in contrast, owing to their loose stomatal management measures, anisohydric plants maintain relatively high stomatal opening even under water shortage conditions, and their STE should decrease under drought conditions (**Figure 1**).

Plant drought resistance strategies can be distinguished by analyzing the changes in functional traits caused by drought (Ingrisch and Bahn, 2018; Hoover et al., 2021). Functional traits are defined as core plant attributes that can strongly influence ecosystem functions and reflect the response of vegetation to environmental changes, including both morphological and physiological characteristics (Fonseca et al., 2000; Markesteijn and Poorter, 2009; Kramp et al., 2022). Morphological traits are the result of long-term adaptations of plants to the characteristics of their growth environment and reflect their ecological niches in the community. Conversely, physiological traits can change rapidly in response to environmental variations and better reflect the drought adaptation ability and mechanisms of plants over a short period (Belluau and Shipley, 2017). Plants often regulate several functional traits to minimize the negative effects of drought. These include (1) increasing water acquisition from the soil; (2) reducing water loss through stomatal regulation; (3) minimizing cellular damage from drought via peroxide scavenging; and (4) enhancing water absorption and retention through the synthesis of molecules that aid in osmotic adjustment (Gupta et al., 2020; Rodrigues et al., 2019). Certain morphological, structural, and growth characteristics also reflect the drought resistance strategies of plants.

For subtropical mixed coniferous and broad-leaved forests in the southwestern karst region of China, angiosperms and gymnosperms that together form the tree layer have been separated for approximately 300 million years (Smith et al., 2010). These plants present many differences in morphology, development, and physiological regulation, which also lead to significant differences in stomatal regulation, water conductivity, and root absorption capacity (Raghavan and Sharma, 1995; Piper et al., 2019). As a result, they have different adaptive abilities and strategies under drought conditions (Dickman et al., 2015; Woodruff et al., 2015), especially during the seedling stage (Raghavan and Sharma, 1995). Species-specific responses to environmental factors during the establishment and seedling stages of a population are considered key features in determining community structure and dynamics; thus, understanding the adaptation abilities and mechanisms of seedlings to environmental changes is highly important for comprehending the potential responses of plant communities in this region to global climate change.

*Platycladus orientalis* (a gymnosperm) and *Broussonetia papyrifera* (an angiosperm) are widely distributed in subtropical China and are often dominant species within the same vegetation communities (Huston, 2004; Lei et al., 2010). This study aimed to investigate functional traits such as water source and consumption, osmotic adjustment and antioxidant capacity, as well as morphological traits of *P. orientalis* and *B. papyrifera* seedlings during continuous drought, for the following purposes:

1. to analyze differences in drought resistance strategies between these two tree species at the seedling stage;
2. to identify indicator traits that distinguish their drought adaptation mechanisms;
3. to provide theoretical insights into methodologies for differentiating plant drought resistance strategies and screening indicator traits.

## 2 Materials and methods

### 2.1 Plant cultivation and trait measurements

The broadleaf tree species *Broussonetia papyrifera* from the Moraceae family and the coniferous tree species *Platycladus orientalis* from the Cupressaceae family were selected as the experimental materials. The entire experiment was conducted in Guiyang, Guizhou Province, China. Seeds were sown in wet perlite and germinated in a greenhouse at 25°C. On the 15th day after germination, healthy seedlings were transplanted into humus soil. The growth environment conditions of the seedlings were as follows: a 12-hour photoperiod, a day/night temperature of 25/16°C, a photosynthetic photon flux density of 400 µmol m^−2^·s^−1^, and a relative humidity of 60–65%. Uniform and vigorous 25 cm tall seedlings were used for this study.

The selected seedlings were planted alone in 20 cm diameter pots with 45 cm deep calcareous yellow soil. Both tree species were divided into treatment and control groups. After the start of the treatment, the pots in the control group were weighed daily and supplemented with appropriate water to replenish the water consumed via evapotranspiration; to simulate prolonged drought, the pots in the treatment group were not watered until the end of the experiment (21 days). Plant height, crown width, net photosynthetic rate (*Pn*) and transpiration rate (*Tr*) were measured every 5 days, and leaf, xylem and soil samples were collected simultaneously to determine the stomatal status, water content, δ^18^O [because moisture in the filling and releasing soil can lead to a vertical gradient of soil H and O isotopes, δ^18^O and δ^2^H in the xylem of plants can be used to calculate or represent the source of soil moisture in plants (Dawson and Ehleringer, 1993; Grossiord et al., 2017)], total soluble sugar content (SS), proline content, thiobarbituric acid reactive substances (TBARS) content and 2,2-diphenyl-picrylhydrazyl (DPPH) radical-scavenging activity.

### 2.2 Stomatal area calculation

Healthy, fully expanded leaves were selected at approximately 11 a.m., and nail polish was evenly applied to the lower surface of the leaves. After the nail polish dried, it was removed with transparent tape. The obtained samples were observed under a microscope, and images were taken. ImageJ was used to analyze the stomatal density and area of the leaves. Finally, the average pore area per stomata was multiplied by the stomatal density to obtain the stomatal area per unit leaf area (Savvides et al., 2012).

### 2.3 Measurement of leaf gas exchange

Healthy, fully developed leaves in the middle layer of the canopy were selected at approximately 11 a.m., and the E and *Pn* of the plants were measured using an LI-6400 (Li-6400, LI-COR). The test was conducted at 25°C, and the photosynthetic photon flux density was 400 μmol m^-2^ s^-1^. In accordance with the mass–area model of *P. orientalis* leaves constructed by Li (1991), the leaf area was calculated by measuring the dry weight of the leaves of the tested part of *P. orientalis* using the following Equation 1.


(1) $$S_{ori}=161\times M_{ori}$$

where S_ori_ and M_ori_ are the leaf area and dry weight of *P. orientalis*, respectively.

### 2.4 Stomatal transpiration efficiency and water use efficiency

Because the LI-6400 cannot directly measure the area of the stoma, the relationship between the stomatal area and transpiration rate of different plants in different environments must be studied using other methods. We calculated the stomatal area of the leaves through graphical analysis, and combined with the Tr measured by LI-6400, the STE was calculated according to the following Equation 2:


(2) STE=E÷SA

The WUE was calculated according to the following Equation 3:


(3) WUE=Pn÷E

where STE is the stomatal transpiration efficiency, E is the transpiration rate, SA is the stoma area, WUE is the water utilization efficiency and *Pn* is the photosynthetic rate.

### 2.5 Oxygen isotope measurements of plants and soil

The plants were irrigated with distilled tap water (δ^18^O = −9.162‰, δ^2^H = −56.958‰). Because plants accumulate water mainly at night, the stems of the plants were collected at 7:00 AM to measure the oxygen isotopes of the xylem water. Soil samples at depths of 10 and 40 cm were also collected simultaneously for determination of the oxygen isotope composition. Soil and plant xylem water were extracted using an LI-2000 water vacuum extraction system (LI-2000, LICA), and the δ^18^O was measured with a continuous-flow isotope ratio mass spectrometer (MAT 253; Thermo Fisher Scientific) according to the method described by Yao et al. (2023).

### 2.6 Xylem water source calculation

Owing to the thin soil layer in the pots, the water sources for each plant’s soil were divided only into surface and deep layers, and soil samples were taken at depths of 10 cm and 35 cm, respectively. After the water oxygen isotope composition in the plant xylem and each soil layer was measured, the water source of the plant in the soil was calculated using a binary linear equation.

### 2.7 Measurement of soluble sugar and proline content

The osmotic adjustment indicators were determined using a soluble sugar content test kit (Comin, China) and a proline content test kit (Comin, China). All the above tests were conducted by spectrophotometry, following the instructions provided with the test kits.

### 2.8 Determination of DPPH radical-scavenging activity

The DPPH radical-scavenging activity was measured and calculated as described by Kontogiorgis and Hadjipavlou-Litina (2004). One gram of plant material was added to 80% methanol and ground into a homogenate in an ice bath. The samples were subsequently centrifuged at 3500 × g for 10 minutes at 4°C, after which the supernatants were collected for analysis. In a test tube, 0.1 mL of the supernatant and 4.9 mL of 0.2 mM 2,2-diphenyl-picrylhydrazyl dissolved in 80% methanol were added to form a reaction system, which was subsequently incubated in the dark at 22°C for 15 minutes. The OD value was subsequently measured at 517 nm. The formula for calculating the DPPH radical-scavenging activity is as follows Equation 4:


(4) $$DPPH\%=(A_{blank}-A_{sample})÷A_{blank}\times 100\%$$

where A_blank_ and A_sample_ are the absorbance values of the blank and sample, respectively.

### 2.9 TBARS content determination

In accordance with the methods of Heath and Packer (1968), the degree of lipid peroxidation was determined by measuring the accumulation concentration of TBARS. Fresh plant material (0.1 g) was ground into a homogenate with 1 mL of 0.1% (w/v) trichloroacetic acid and then centrifuged at 10,000 × g for 20 minutes. Subsequently, 0.5 mL of the supernatant was mixed with 1 mL of 0.5% (w/v) TBA (20% TCA) and incubated in a 98°C water bath for 30 min. After incubation, the mixture was placed in an ice bath for 10 min and centrifuged again at 10,000 × g for 5 min. The OD values of the supernatants were measured at 450, 532, and 600 nm. The content of TBARS was calculated using the following Equation 5:


(5) $$TBARS=6.45(OD_{532}-OD_{600})-0.56OD_{450}$$

### 2.10 Water content determination

The leaf and soil water contents (LWC and SWC, respectively) were measured as described by Yao et al. (2023). The fresh mass of the leaves was measured immediately after collection and cleaning. The leaves were subsequently dried at 65 °C until a constant weight was reached, after which the dry mass was recorded. The water content of the leaves was calculated using the following Equation 6:


(6) (LFM−LDM)/LFM

where LDM and LFM are the dry mass and fresh mass of leaves, respectively.

Following collection, the soil sample was sieved through a 1 mm mesh, and its fresh mass was measured immediately. The sample was then dried at 65 °C until a constant weight was attained, and the dry mass was determined accordingly. The SWC was calculated by the following Equation 7:


(7) SWC=(SFM−SDM)/SDM

where SDM and SFM are the dry mass and fresh mass of the soil, respectively.

### 2.11 Crown width and height measurements

The widest part of the tree crown was selected. Every 45° rotation was measured once through the cross-section of the tree crown axis, for a total of 4 measurements. The average of the 4 measurements was taken as the crown width value. The tree height was directly measured with a tape measure.

### 2.12 Chlorophyll content determination

The chlorophyll content was measured as described by Sartory and Grobbelaar (1984). Leaves (0.1 g) were weighed and ground into fragments in liquid nitrogen. Ten millilitres of 80% acetone was added, and the mixture was mixed well and centrifuged at 4,000 r·min^−1^ for 15 minutes. Then, 1 mL of the supernatant was removed, and 3 mL of acetone was added. The OD values at 645 nm and 663 nm were measured using a spectrophotometer (BlueStar, LabTech, USA).

The chl a content was calculated according to the following Equation 8:


(8) $$Chl\;a=12.7OD_{663}-2.69OD_{645}$$

The content of chl b was calculated according to the following Equation 9:


(9) $$Chl\;b=22.9OD_{645}-4.64OD_{663}$$

### 2.13 Abscisic acid content

Fresh leaves (0.1 g) were washed with 10 mL of distilled water, liquid nitrogen was added, and the mixture was ground into a homogenate. A total of 450 µL of 100% methanol was added, and the mixture was centrifuged at 800 × g at 4°C for 10 minutes. The supernatant was collected for ABA content determination. The ABA content was determined using an ABA ELISA kit (Cusabo Technology LLC, Wuhan, China) following the manufacturer’s instructions. The absorbance of ABA was measured at 450 nm using a microplate spectrophotometer (Synergy H1/H1MFD, BioTek, USA). The ABA content is expressed as ng g^-1^ FW.

### 2.14 Determination of xylem water content

At 6 a.m., a 3 cm long stem was cut from the base of each plant. The bark was peeled off, and the fresh weight was determined. The stem segment was then dried at 60°C until a constant weight was reached, after which the dry weight was determined. The XWC was calculated using the following Equation 10:


(10) $$XWC=(M_{f}-M_{d})÷M_{d}$$

where M_d_ and M_f_ are the dry mass and fresh mass of the xylem, respectively.

### 2.15 Detection of xylem water by magnetic resonance imaging

After 18 days of the experiment, the young *B. papyrifera* and *P. orientalis* seedlings were removed from the soil to completely cut off their water supply. Five days later, the water status of the stems was detected using a magnetic resonance imaging system (Skyra 3.0T, Siemens, Germany). Severing the xylem will cause the water in the xylem vessels to rapidly contract radially under atmospheric pressure. Therefore, the branches were immersed in water when cut to avoid disrupting the water distribution characteristics within the vessels. The cut branches were wrapped in cling film and then in insulating aluminum foil and moved to the MRI laboratory as soon as possible for testing at a location as far from the cut as possible. Owing to the high sensitivity and strong signal of hydrogen nuclei in nuclear magnetic resonance, hydrogen nuclei were selected as the imaging element to display the water distribution in the biological body. The differences in hydrogen nucleus density and relaxation times at T1 and T2 are the main physical basis for the use of MRI in diagnosis and detection. Xylem water imaging was performed using a magnetic resonance imaging system (Skyra 3.0T, Siemens, Germany). Assistance with the parameter settings and the operation process for MRI detection was provided by the radiology department of Tongren People’s Hospital in Guizhou Province.

### 2.16 Data analysis

Five replicates were used for all measurements except the isotope composition measurement (three replicates). Principal component analysis (PCA) was performed to assess the drought resistance strategies of the plants via PCA v1. 50 of OriginPro 2021 (OriginLab Inc.). Pearson’s correlations between the soil water content and plant traits and between the traits of different plants were performed with Correlation Plot v1.31 of OriginPro 2021 (OriginLab Inc.). A polynomial fit model was used to explore the differences (*p* < 0.05) in trait changes with soil water content between *P. orientalis* and *B. papyrifera* using Simple Fit v3.10 of OriginPro 2021 (OriginLab Inc.). One-way ANOVA followed by Duncan’s multiple range test was performed in SPSS 25.0 (SPSS Inc.).

## 3 Results

### 3.1 Distinguishing drought resistance strategies through principal component analysis

**Figure 2:**
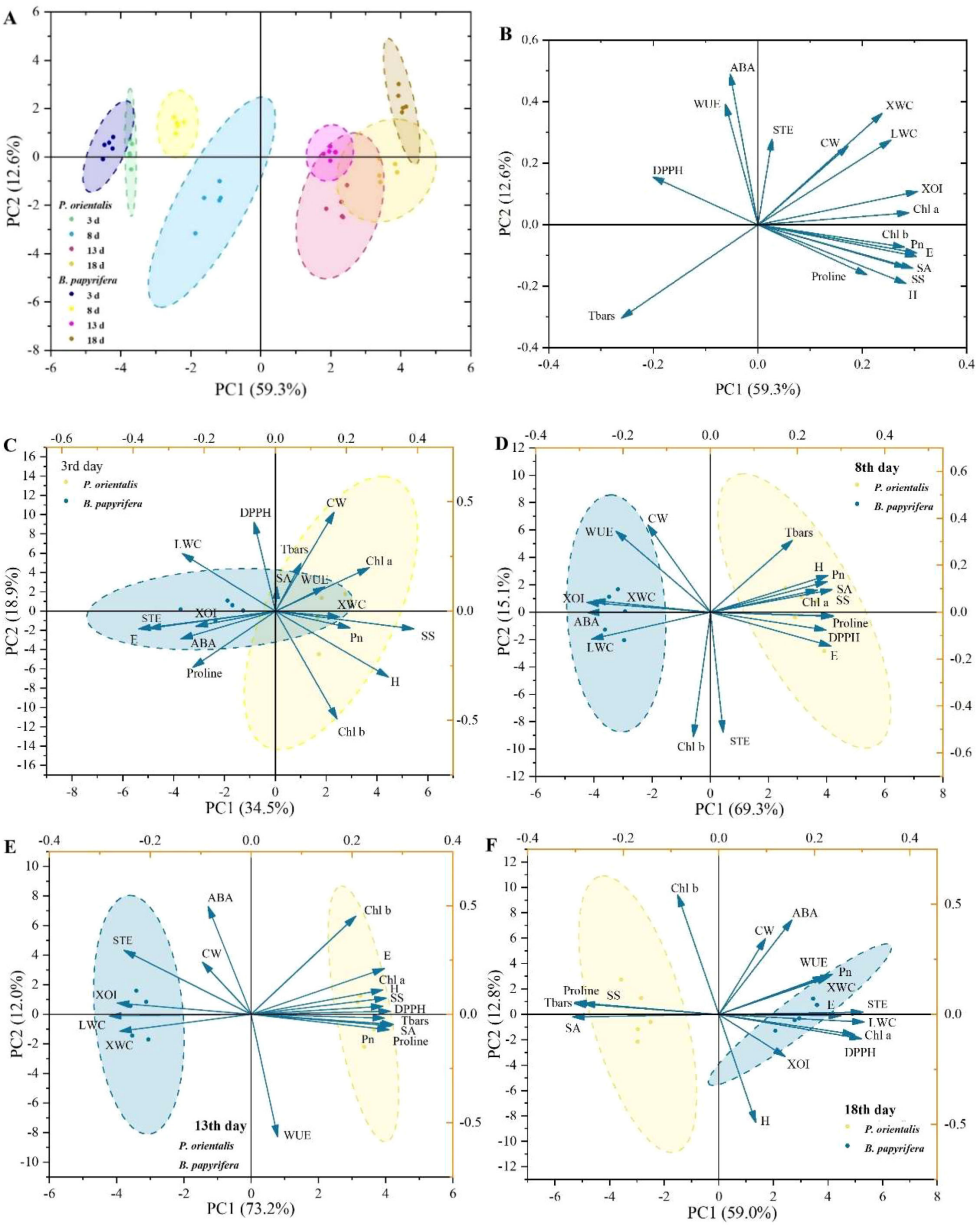
PCA of the functional traits of *P. orientalis* and *B*. *papyrifera* for distinguishing drought resistance strategies. **(A)** Score plot of traits. **(B)** Loading plot of traits. Score and loading plots of traits on the **(C)** 3rd day, **(D)** 8th day, **(E)** 13th day, and **(F)** 18th day. ABA, abscisic acid; CW, crown width; DPPH, 2,2-diphenyl-picrylhydrazyl scavenging ability; E, transpiration rate; H, height; LWC, leaf water content; *P*n, net photosynthetic rate; SA, stomatal area; SOD, superoxide dismutase; SS, soluble sugar; STE, stomatal transpiration efficiency; TBARS, thiobarbituric acid reactive substance; WUE, water use efficiency; XOI, xylem δ^18^O; XWC, xylem water content.

On the basis of the various traits (**Supplementary Figure S1**) associated with the responses of plants to drought stress, PCA was used to compare the drought resistance strategies of the two tree species. As shown in **Figure 2A**, the plants under different treatment times exhibited significantly different responses, indicating that the continuous reduction in soil moisture due to the increase in drought treatment time was the most important factor determining the growth, physiological conditions and responses of the plants. The distribution of the subsets of the two plants in the PCA coordinate system revealed differences in drought response strategies between *P. orientalis* and *B. papyrifera*. Overall (**Figure 2B**), PC1 (59.3%) explained the main differences between *P. orientalis* and *B. papyrifera*. Traits related to moisture characteristics (XOI and E) and photosynthetic characteristics (Chl a, Chl b and Pn) had high loadings on PC1; ABA, STE and WUE had high contributions to PC2. **Figure 2C–F** shows that during the first 13 days, the separation of the drought response characteristics (drought resistance strategies) of the two plants became increasingly obvious; however, on the 18th day, the subsets representing the drought resistance strategies of the two plants moved closer again. Notably, on the 8th and 13th days, when the subsets of the two plants were significantly separated, the degree of explanation of PC1 for the differences between the two tree species was very high, reaching 69.3% and 73.2%, respectively. Among all 17 traits, 12 (loadings, 0.24–0.29) and 13 (loadings, 0.25–0.29) had strong and similar loadings on PC1.

### 3.2 Screening indicator traits through correlation analysis

**Figure 3:**
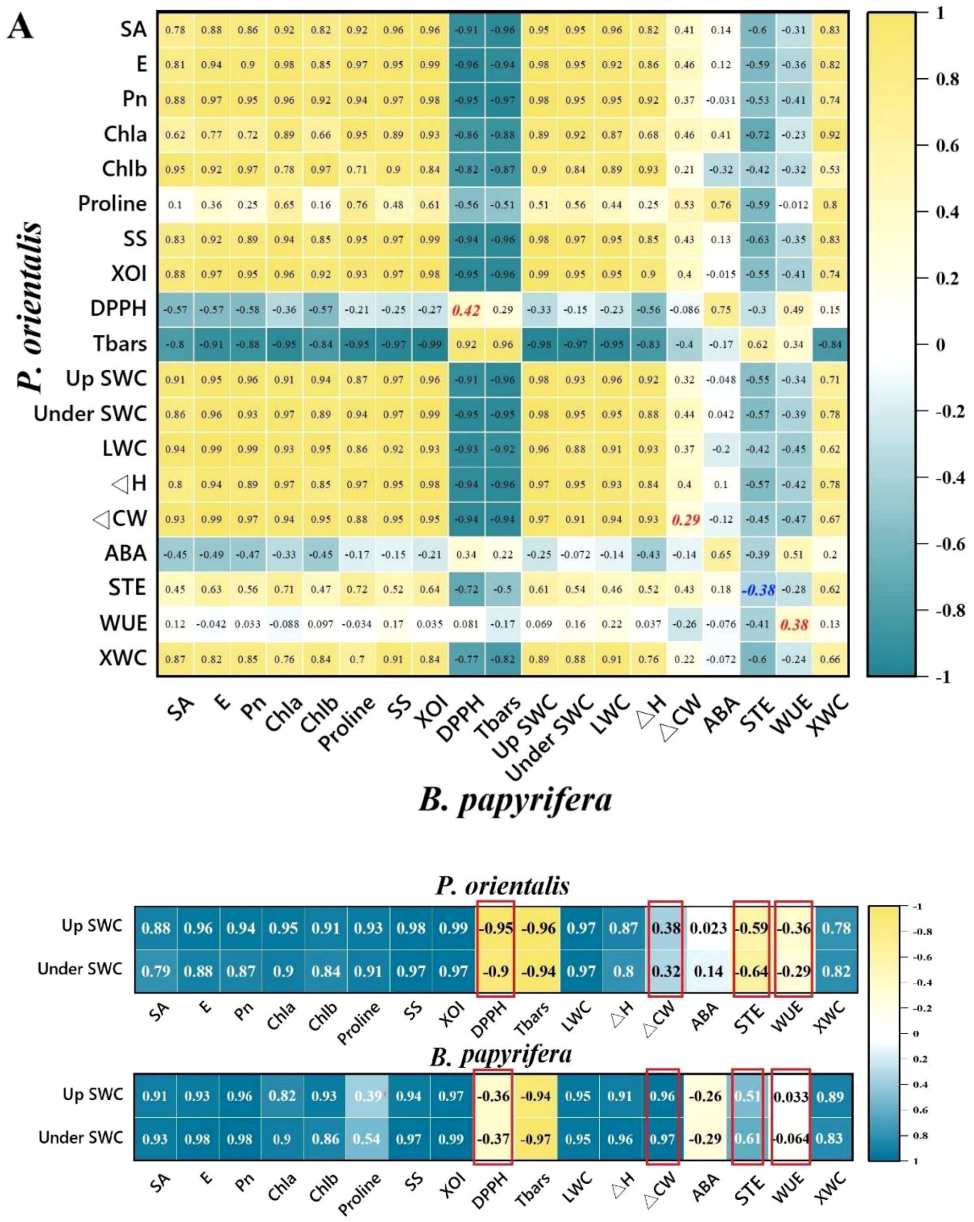
Indicator traits were screened through correlation analysis. **(A)** Correlations between the same traits in *P. orientalis* and *B*. *papyrifera* under drought treatment. **(B)** Correlations between the traits of the plants and the soil water content. See the abbreviations used in **Figure 2**.

We attempted to identify indicator traits that distinguish the drought resistance strategies of plants by analyzing the correlation between plant traits and soil moisture, as well as the correlation between the same traits of two plant species under the same drought treatment. The results of the Pearson correlation analysis (**Figure 3A**) indicated that under the same drought treatment conditions, the correlations between the changes in the ΔCW, STE, and WUE of the two plants were poor (*r* < 0.40, *p* < 0.05). Among them, the STE values of the two plants were weakly negatively correlated. The correlation between the changes in DPPH of the two plants was also low (*r* = 0.42). As shown in **Figure 3B**, Pearson correlation analysis indicated that many plant traits were highly correlated with the moisture content of the upper soil layer. For the 4 traits selected through the correlation analysis between tree species, the DPPH, ΔCW, STE and WUE of *P. orientalis* were highly negatively correlated, weakly positively correlated, moderately negatively correlated and weakly correlated with soil moisture, respectively, whereas the DPPH, ΔCW, STE and WUE of *B. papyrifera* were weakly negatively correlated, highly positively correlated, moderately positively correlated and weakly correlated with soil moisture, respectively.

### 3.3 Interspecific differences in plant DPPH, ΔCW, STE and WUE

**Figure 4:**
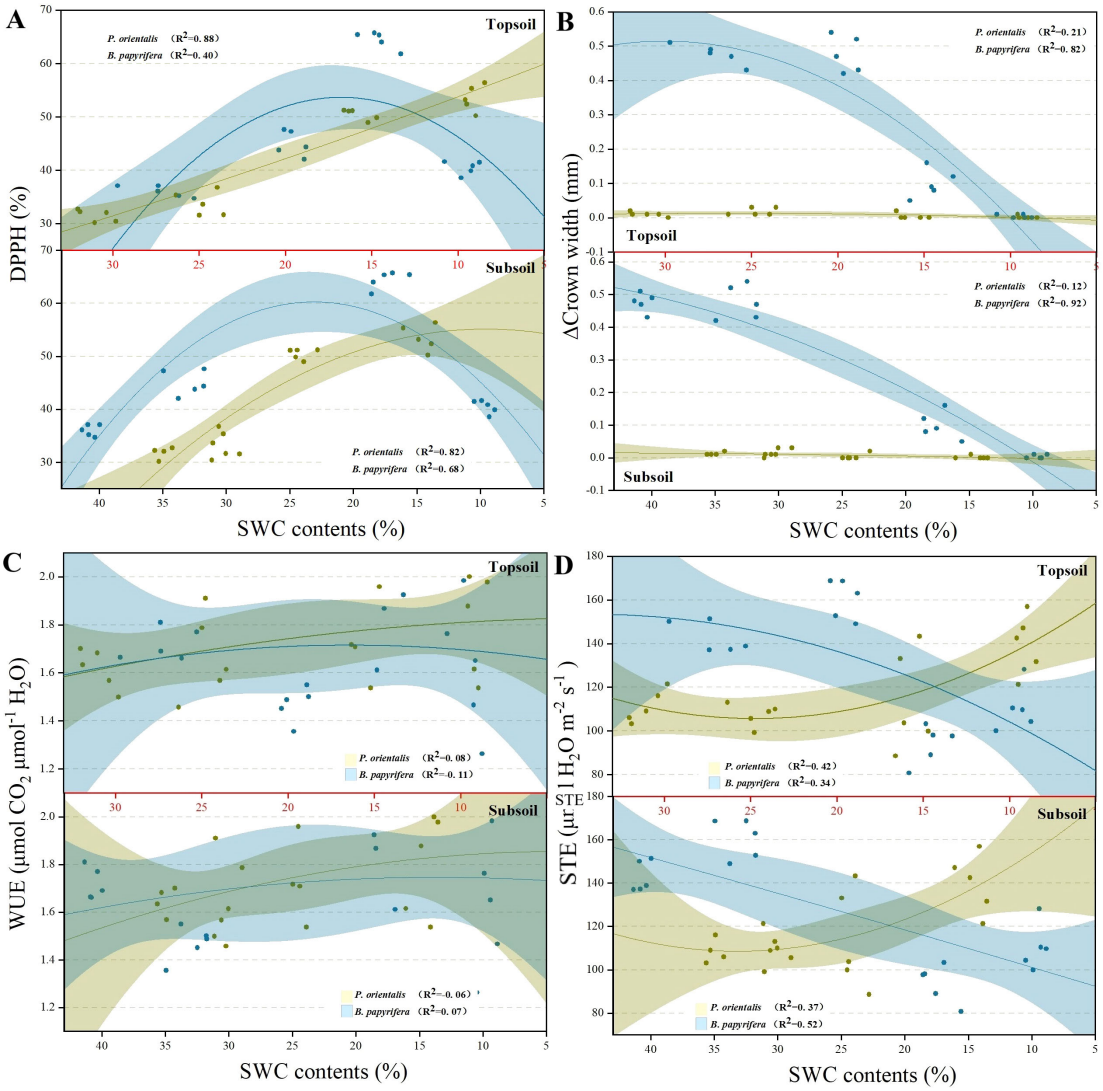
The changing trends of **(A)** DPPH, **(B)** CW, **(C)** WUE and **(D)** STE in plants under persistent drought. See the abbreviations used in **Figure 2**.

**Figure 4A** shows that the growth of the crown of *P. orientalis* almost stopped at the beginning of the drought treatment. **Figure 4B** shows that the DPPH activity of *B. papyrifera* increased sharply but then decreased sharply with decreasing soil moisture, whereas the DPPH scavenging ability of *P. orientalis* increased with increasing drought stress. **Figure 4C** shows that the STE of *P. orientalis* tended to increase during the drought treatment, whereas the STE of *B. papyrifera* decreased significantly. **Figure 4D** shows that the WUE of *P. orientalis* increased significantly with decreasing soil moisture content, whereas the WUE of *B. papyrifera* increased significantly only in the middle stage of the drought treatment.

### 3.4 Xylem moisture characteristics of *P. orientalis* and *B. papyrifera*

**Figure 5:**
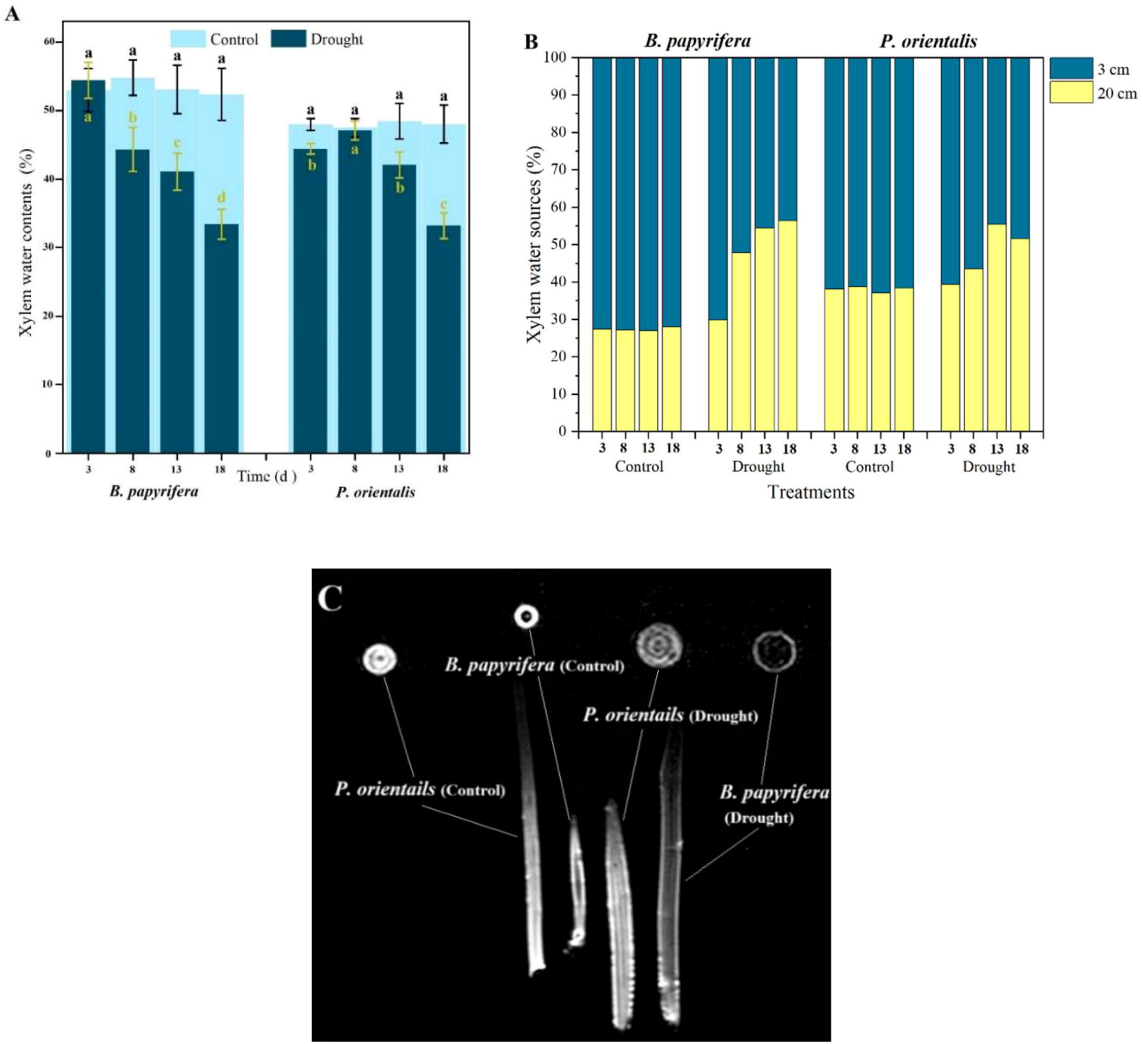
The moisture status of the xylem. **(A)** Xylem water sources of plants. **(B)** The water contents of the xylem at predawn. **(C)** Xylem water detected by magnetic resonance imaging.

As shown in **Figure 5A**, compared with *B. papyrifera*, *P. orientalis* presented a greater ability to restore xylem moisture by absorbing water at night. By the 13th day before dawn, the water content in the xylem was still close to 90% of that in the xylem of the control plants.

Testing the oxygen isotope composition of the xylem in the plants (**Supplementary Figure S1L**) and the soil water δ^18^O values in different soil layers (**Supplementary Figure S4**) enabled the proportion of water absorbed by plants from different soil layers to be calculated (**Figure 5B**). The results indicated that under well-watered conditions, both *B. papyrifera* and *P. orientalis* mainly absorbed water from the upper soil layer. As the duration of drought treatment increased, the utilization of water from the subsoil layer increased in both species in the treatment groups.

After the treatment experiment, the *B. papyrifera* and *P. orientalis* seedlings were removed from the soil to completely cut off their water source. Five days later, the water status of the xylem was imaged by nuclear magnetic resonance. As shown in **Figure 1**, even though the plants had died from drought, the xylem of *P. orientalis* still presented a strong water signal, whereas the water signal of the xylem of *B. papyrifera* was very weak (**Figure 5C**).

## 4 Discussion

### 4.1 PCA based on drought response traits can distinguish the drought resistance strategies of plants

It is generally believed that in arid environments, gymnosperms tend to adopt a drought avoidance strategy, whereas angiosperms tend to adopt a drought tolerance strategy. PCA can identify distinctive features among different research samples and screen out indicator traits. The results revealed that *P. orientalis* and *B. papyrifera* are well separated in the PCA coordinate system (**Figures 2D, E**), indicating that PCA based on functional traits can distinguish the drought resistance strategies of plants.

PCA of various drought response traits of plants revealed that as the duration of drought events increased, the differences in drought resistance strategies between *B. papyrifera* and *P. orientalis* became more pronounced; however, in the later stage of treatment, the differences in their drought resistance strategies diminished (**Figures 2C–F**). The PCA results indicate that the explanatory power of PCs for total variation decreases in the later stage of treatment, and the loadings of many traits on PCs also decrease. These findings suggest that in the later stage of drought treatment, more drought response traits of *B. papyrifera* and *P. orientalis* undergo similar changes. Kramp et al. (2022) reported that as the severity of drought intensifies, plants’ drought resistance strategies shift from tolerance to avoidance. We do not believe this is an active choice of drought resistance strategies by plants but, rather, a result of severe drought stress, which forces different plant species to adopt more stringent water control measures, allocate more energy to the removal of peroxides, and reduce investment in growth. This pattern also poses challenges to the analysis of plant drought resistance strategies and the selection of trait variables. Choosing an appropriate time point at which to distinguish the drought resistance strategies of plants—or of select traits that are less affected by drought severity—is highly important for the study of plant drought resistance strategies.

### 4.2 Correlations of the same trait between species can be used to screen potential indicator traits

When plants are subjected to drought stress, avoidance and drought tolerance strategies often coexist. However, some evolutionary divergences and physiological metabolic trade-offs lead to significant differences in drought adaptation characteristics among different plant species (Hendrickson et al., 2025). Through PCA and correlation analysis, we aimed to identify indicator traits that can facilitate the rapid identification of the drought resistance strategies used by different species.

First, we attempted to screen for indicator traits that distinguish the drought strategies of different species through PCA. The results indicated that physiological traits such as XOI, photosynthetic function traits (Chl a, Chl b and Pn), E, ABA, STE and WUE strongly contributed to the PCs, suggesting that the differences in stomatal regulation, photosynthetic capacity and the ability to adjust to a changing water ecological niches play significant roles in distinguishing different drought resistance strategies. These results further verified that hydraulic failure and carbon starvation were the two most important factors leading to plant death in drought environments, as proposed in the isohydric–anisohydric model. Generally, plants that adopt a drought tolerance strategy are believed to have stronger osmotic adjustment and antioxidant capabilities (Pausas et al., 2014); however, PCA revealed that proline and SS, which reflect the osmotic adjustment ability of plants, and DPPH, which reflects the peroxide scavenging ability of plants, have relatively small contributions to the PCs. Additionally, plants that adopt drought avoidance strategies and those that adopt drought tolerance strategies present significant differences in their growth rates. Coniferous plants adopt a conservative resource utilization strategy and have a slower growth rate (DeSoto et al., 2020; Piper et al., 2019), whereas broad-leaved plants adopt an acquisition-type resource utilization strategy and have a faster growth rate under suitable conditions. However, the PCA results revealed that growth traits made relatively small contributions to the PCs and were not effective indicators for distinguishing drought strategies, which might be due to the short duration of the experiment. Notably, in both the overall analysis of the entire experimental process and the PCA of the two plant subsets on the 8th and 13th days, when significant separation occurred, more than half of the traits had strong and similar loadings in the PCs, indicating that the responses of plants to drought and their drought strategies are reflected in various aspects of growth and physiology; thus, it is difficult to screen out one or two traits through PCA to distinguish the drought strategies or adaptation characteristics of plants.

Second, we screened the indicator traits through correlation analysis. We believe that the correlation analysis of different plant functional traits during the drought treatment process can better reflect the differences in drought adaptation strategies among species. That is, the greater the positive correlation of a certain trait between two plants under the same drought treatment process is, the more similar their adaptation to drought; conversely, the lower the positive correlation, or even a negative correlation, of a certain trait during the drought treatment process is, the greater the difference in their adaptation to drought. The traits with poor correlations between the two species in the experiment included STE, WUE, ΔCW and DPPH, which reflected their differences in drought adaptation in terms of stomatal control, photosynthetic efficiency, growth characteristics and antioxidant capacity. Interestingly, these traits with low correlations were all derived or comprehensive metrics, which also indicated, to some extent, that it might be difficult to distinguish the differences in drought strategies of tree species on the basis of only one or two basic traits. Notably, the STEs of the two tree species were negatively correlated, indicating a significant difference in stomatal regulation between *B. papyrifera* and *P. orientalis*.

Bandurska (2022) proposed that plant adaptations to drought can be classified into adaptation and acclimation. Adaptation refers to the characteristics acquired by organisms through long-term evolution, whereas acclimation can be regarded as the adaptation of plants to the environment through plasticity. Adaptation traits seem to have greater potential to be indicator traits that distinguish the drought resistance strategies of species without varying with the degree of drought. The results of the correlation analysis between plants and soil moisture indicate that some indicators that are weakly correlated with soil moisture changes, such as the ΔCW, STE, and WUE of *P. orientalis* and the DPPH, STE, and WUE of *B. papyrifera*, are potential traits indicating adaptation. However, when the time variation curves of these indicators are combined, the reasons for their low correlation with soil moisture changes are not consistent. The low correlation between the CW of *P. orientalis* and soil moisture changes is due to the slow growth characteristics of this species; ABA may accumulate rapidly when water slightly decreases, thus losing its linear correlation with water availability (Verslues and Zhu, 2005). In contrast, the poor correlation between the DPPH of *B. papyrifera* and soil moisture changes is due to its rapid growth in the early stage and subsequent decline due to metabolic imbalance of the organism under long-term drought stress, whereas the low correlations between the STE and WUE and soil moisture changes are likely related to the complex correlations between their original indicators and soil moisture changes. Therefore, it is difficult to distinguish the differences in drought resistance strategies among different plant species on the basis of the correlations between plant trait changes and soil moisture.

### 4.3 Interspecific differences in plant DPPH, CW, WUE and STE

Under drought treatment, the growth of the crown of *P. orientalis* stopped very early, whereas the width of the crown of *B. papyrifera* maintained a certain level of growth. This difference was mainly due to the earlier closure of stomata in *P. orientalis*, which weakened photosynthesis. In addition, the differences in resource acquisition and utilization strategies between gymnosperms and angiosperms led to different growth rates. The trend of DPPH changes indicated that *B. papyrifera* relies on DPPH to protect plant cells from peroxide damage under weak drought stress, but the DPPH-scavenging capacity decreases in the later stage of drought stress due to an imbalance in overall metabolic status. *P. orientalis*, which tends towards isohydric behavior, reduces the production of peroxides and their damage to cells because of its better water retention capacity. Many experiments have shown that the WUE of plants increases under drought conditions (Jucker et al., 2017). The experimental results of the present study confirmed this view. In the later stage of drought treatment, the WUE of *B. papyrifera* was significantly lower than that of the control group, and the STE of *P. orientalis* was significantly greater than that of the control group, which may be related to the physiological disorders caused by severe drought treatment. The opposite trends in the STEs of *B. papyrifera* and *P. orientalis* during drought treatment reflected the differences in their stomatal management strategies under drought conditions. The trend of STE in *P. orientalis* can likely be attributed to its strict stomatal management measures, confirming the view that gymnosperms tend towards isohydric behavior, as reported by Martín-Gómez et al. (2017) and Jiménez-Castillo et al. (2022), whereas the significant decrease in the STE of *B. papyrifera* indicates that it is characterized by anisohydric behavior. However, the water loss caused by evaporation from the leaf surface may have interfered with the results, especially by increasing the STE of *P. orientalis* in the later stage of drought treatment. Compared with those of DPPH, CW and WUE, the STEs of the two plants presented more obvious differences in trends under drought treatment. We believe that the STE can be used as an indicator to distinguish the drought resistance strategies of plant species or at least to determine their position along the isohydric–anisohydric spectrum. However, few studies have simultaneously measured the stomatal area and transpiration rate, and additional investigations of different tree species are needed to further verify the reliability of the STE as an indicator trait for differentiating plant drought resistance strategies.

### 4.4 The xylem water content of dead plants can reveal differences in plant drought resistance strategies but has limited applicability

Xylem is an important tissue for water transportation and storage in plants, so we sought to further understand the differences in the water characteristics of the xylem between the two plant species studied. Plants often replenish their tissues with water at night (Moreno et al., 2024). The amount of water replenished by *B. papyrifera* at night decreases as the duration of drought increases, whereas the water replenishment ability of *P. orientalis* is relatively greater (**Figure 5A**). Bartelheimer et al. (2010) suggested that most plants tend to occupy optimal and similar ecological niches when grown in monocultures. However, when facing interspecific competition or environmental changes, plants can change their effective rooting depth to obtain more water (Fan et al., 2017). Under drought treatment, both *B. papyrifera* and *P. orientalis* alleviate water deficiency by increasing water uptake from the lower soil layer (**Figure 5B**). The results of Brodribb and McAdam (2013) indicated that under long-term drought conditions, the stomatal behavior of plants shifts from isohydric to anisohydric. However, the results of nuclear magnetic resonance tests on the stems of the plants in the present study revealed that even when *P. orientalis* died in a drought environment (**Supplementary Figure S5**), a considerable amount of water was still retained in the xylem, demonstrating typical isohydric behavior (**Figure 5C**). Therefore, the water content in the xylem of dead plants can serve as an indicator of the drought resistance strategies of plants. However, owing to the high cost and immobility of nuclear magnetic resonance instruments, the use of xylem water to distinguish the differences in the drought resistance strategies of plants is difficult to apply widely. The cause of plant death was likely carbon starvation. However, the rate of SS consumption in the leaves of *P. orientalis* was not greater than that in the leaves of *B. papyrifera*, possibly because the amount of SS stored in the bodies of the plants was relatively small, regardless of whether they were *P. orientalis* or *B. papyrifera*. This finding also suggests that at the seedling stage, isohydric plants may be more vulnerable than anisohydric plants are to the threat of drought. Notably, isohydric plants die from carbon starvation rather than dehydration. However, the adaptive significance and evolutionary cause of this phenomenon are not yet known.

## Data Availability

The original contributions presented in the study are included in the article/**Supplementary Material**. Further inquiries can be directed to the corresponding author/s.
