# Supplementary material for: Distinguishing drought resistance strategies and identifying indicator traits of Platycladus orientalis and Broussonetia papyrifera
Source: Front Plant Sci. 2025 Aug 20;16:1644756. doi: 10.3389/fpls.2025.1644756 (PMC12405272; doi:10.3389/fpls.2025.1644756)
Supplement: Supplementary file 1 [file Table1.docx]

**^[[1]](#footnote-1)^Supplementary figures**


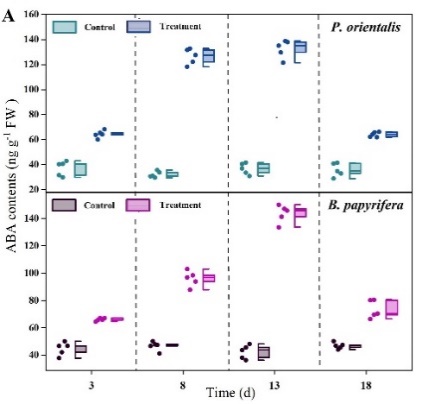

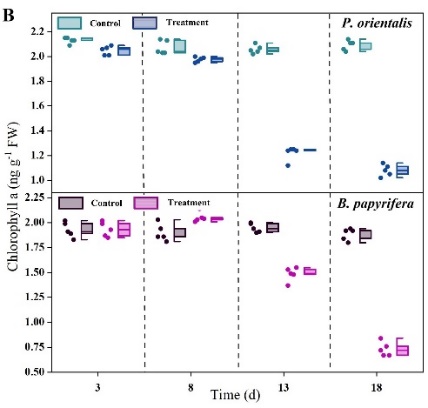

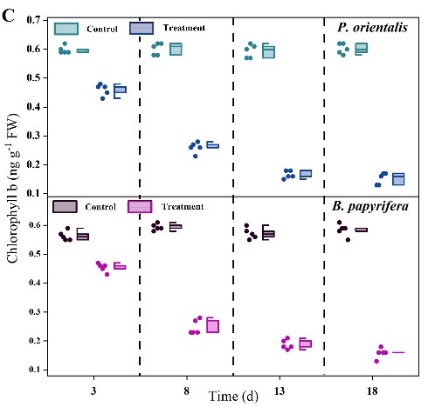

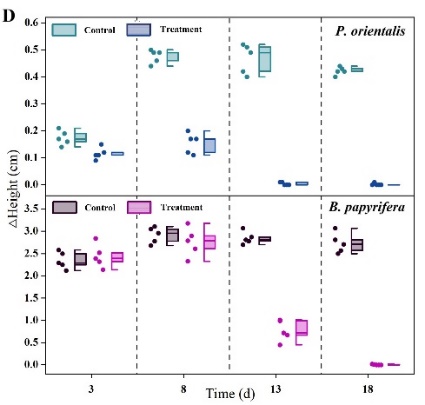

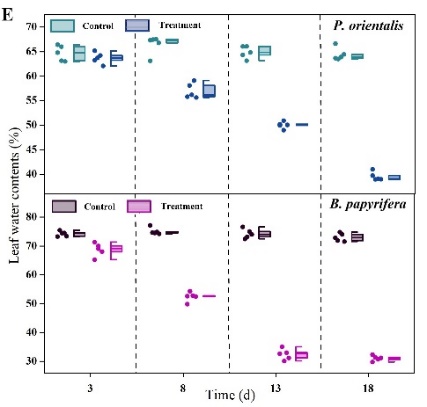

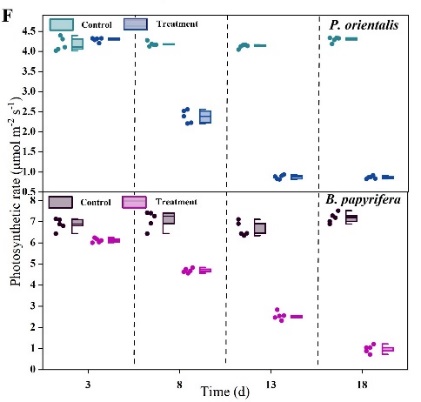

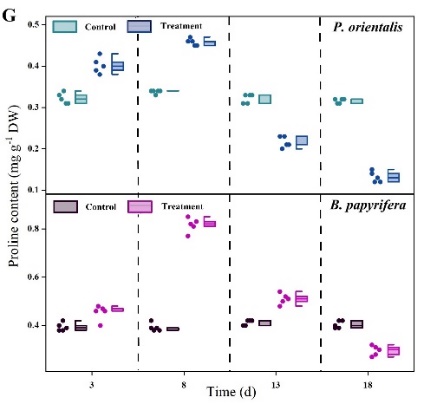

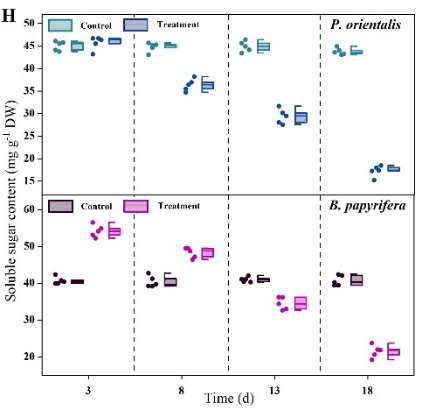

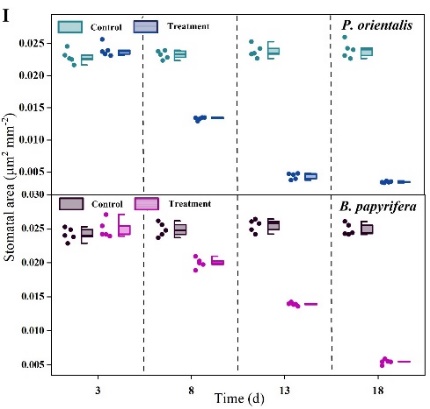

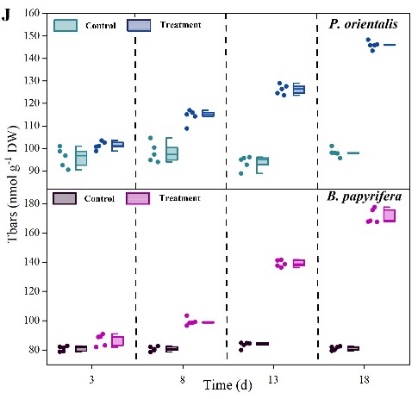

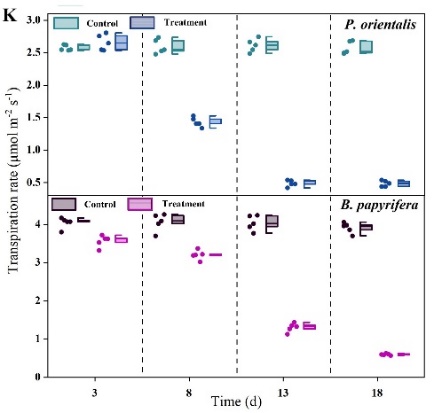

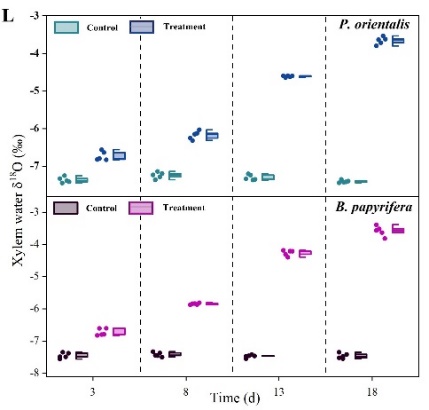


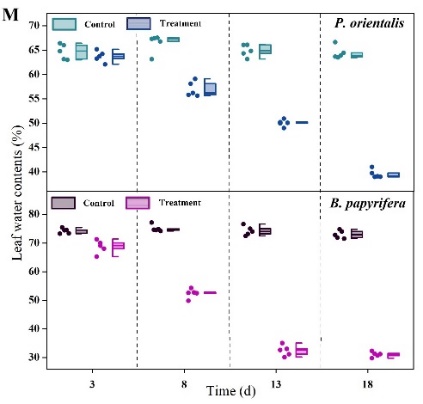


Figure S1

(A~M) Traits of *P. orientalis* and *B. papyrifera* used for PCA analysis.


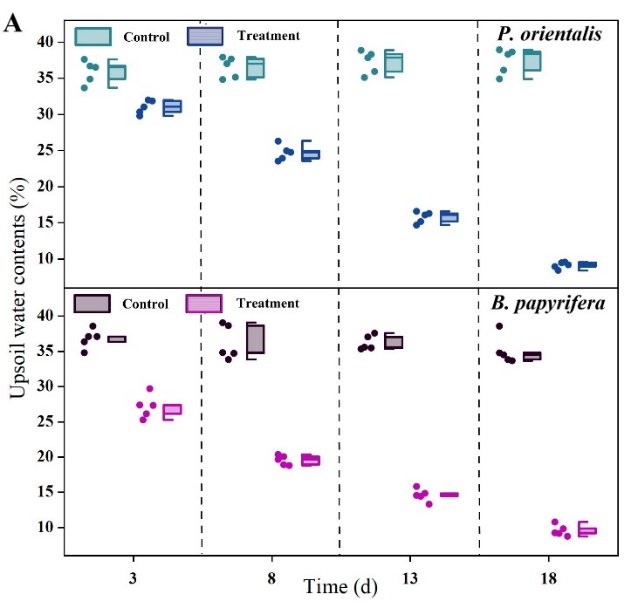

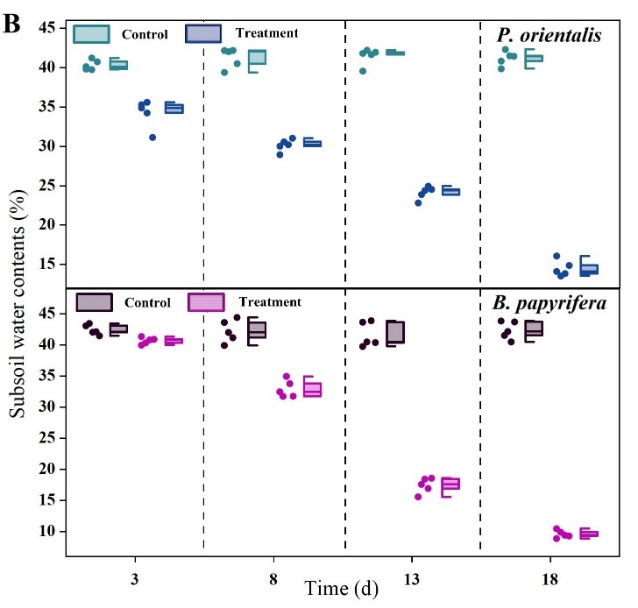


Figure S2

(A) Upsoil and (B) subsoil water contents.

**
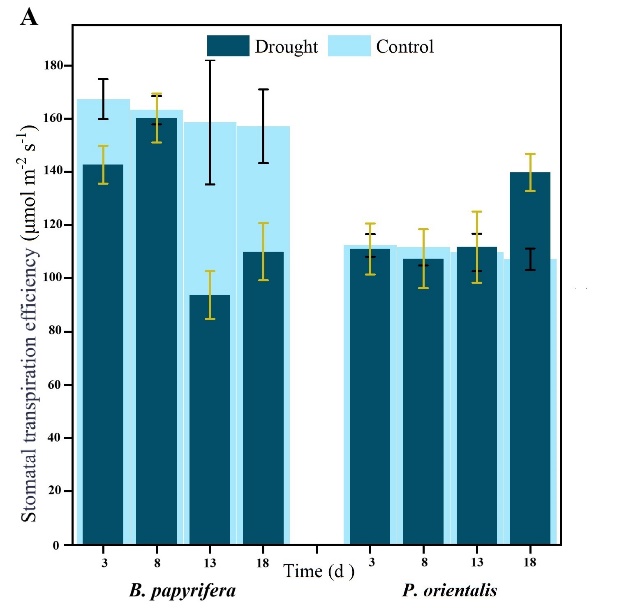

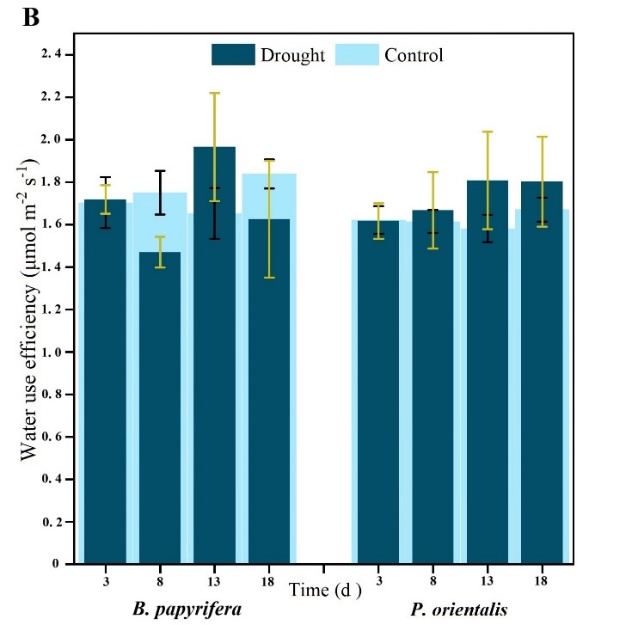
**

**
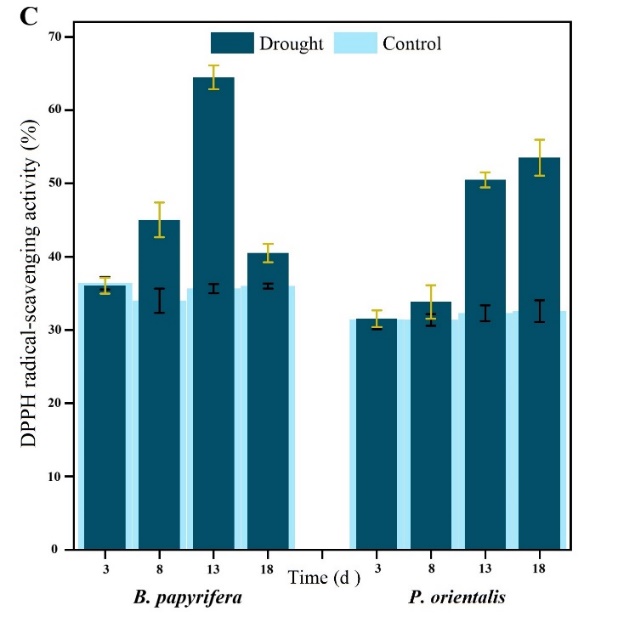

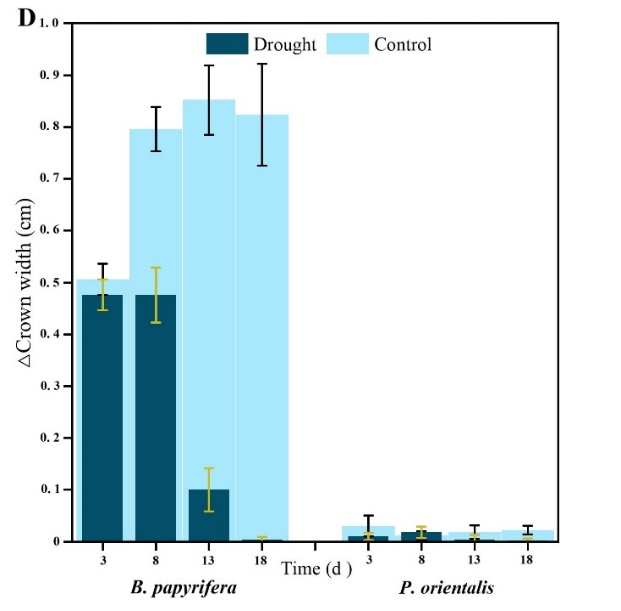
**

Figure S3

(A) STE, (B) WUE, (C) DPPH and (D) ΔCW of *P. orientalis* and *B. papyrifera*.


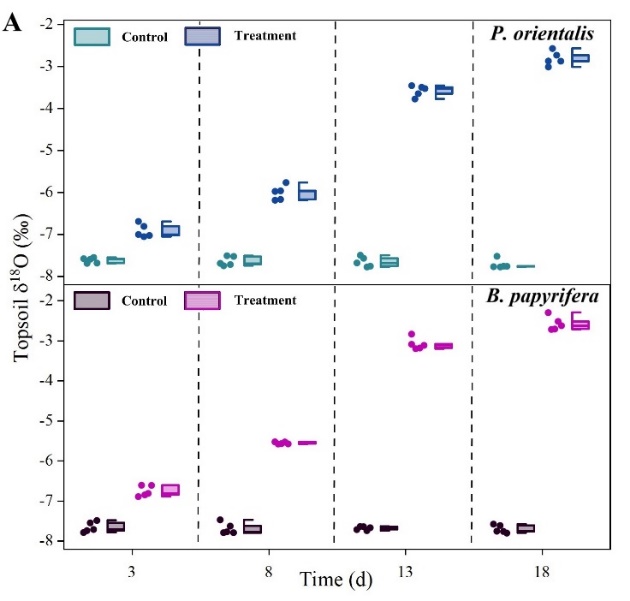

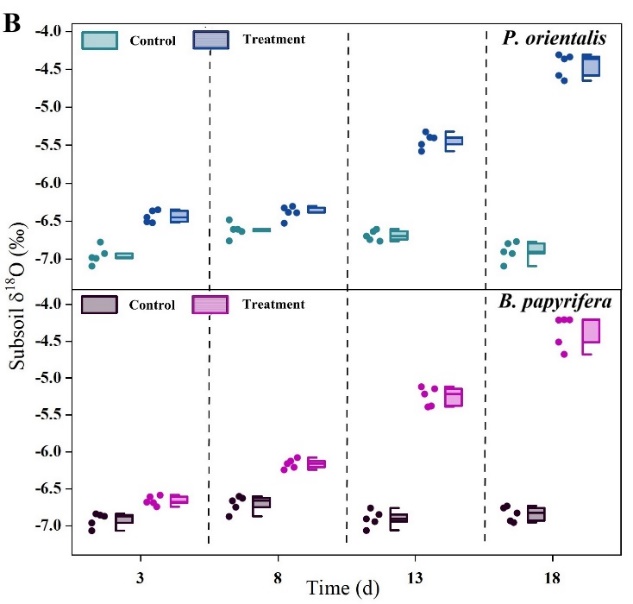


Figure S4

Mean soil water δ^18^O values of (A) upsoil and (B) subsoil under different treatments.


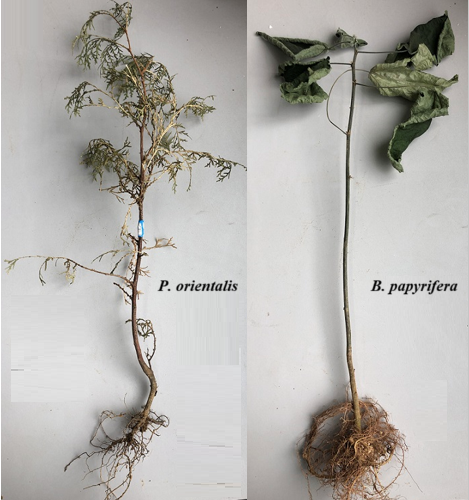


Figure S5

*P. orientalis* and *B. papyrifera* died due to dehydration.

1. [↑](#footnote-ref-1)
